# Written survey on recently deceased patients in germany and switzerland: how do general practitioners see their role?

**DOI:** 10.1186/s12913-016-1257-1

**Published:** 2016-01-20

**Authors:** Rieke Schnakenberg, Adrian Goeldlin, Christina Boehm-Stiel, Markus Bleckwenn, Klaus Weckbecker, Lukas Radbruch

**Affiliations:** 1 Department of General Practice and Family Medicine, University Medical Center Friedrich-Wilhelms-University Bonn, Sigmund-Freud-Str. 25, 53127 Bonn, Germany; 2 Institute of General Practice, University of Bern, Gesellschaftsstrasse 49, CH-3012 Bern, Switzerland; 3 Department of Palliative Medicine, University Medical Center Friedrich-Wilhelms-University Bonn, Sigmund-Freud-Str. 25, 53127 Bonn, Germany

## Abstract

**Background:**

General practitioners (GPs) play an important role in end-of-life care due to their proximity to the patient’s dwelling-place and their contact to relatives and other care providers.

**Methods:**

In order to get a better understanding of the role which the GP sees him- or herself as playing in end-of-life care and which care their dying patients get, we conducted this written survey. It asked questions about the most recently deceased patient of each physician. The questionnaire was sent to 1,201 GPs in southern North Rhine-Westphalia (Germany) and the Canton of Bern (Switzerland).

**Results:**

Response rate was 27.5 % (*n* = 330). The average age of responding physicians was 54.5 years (range: 34–76; standard derivation: 7.4), 68 % of them were male and 45 % worked alone in their practice. Primary outcome measures of this observational study are the characteristics of recently deceased patients as well as their care and the involvement of other professional caregivers. Almost half of the most recently deceased patients had cancer. Only 3 to 16 % of all deceased suffered from severe levels of pain, nausea, dyspnea or emesis. More than 80 % of the doctors considered themselves to be an indispensable part of their patient’s end-of-life care. Almost 90 % of the doctors were in contact with the patient’s family and 50 % with the responsible nursing service. The majority of the GPs had taken over the coordination of care and cooperation with other attending physicians.

**Conclusion:**

The study confirms the relevance of caring for dying patients in GPs work and provides an important insight into their perception of their own role.

## Background

The focus of end-of-life care and especially palliative care is shifting increasingly from specialist inpatient services towards home care [1]. The predominant reason for this is the wish of many people to stay at home until the time of death [2]. Respecting this wish means providing end-of-life care at home according to best medical practice.

In outpatient palliative care settings the evidence supports specialized palliative care services: a Cochrane systematic review based on 37,561 patients and 4042 family caregivers shows that the chance of dying at home increases more than twofold by the employment of home palliative care compared to standard care and that the symptoms are better controlled [3].

Providing specialized palliative care programs in outpatient settings for entire regions seems to be promising. E.g. in Norway a regional outpatient palliative care program led in a cluster-randomized trial to more patients dying at home [4] and to more satisfaction in close family members [5]. Yet, they could not prove a significant improvement in patients’ quality of life [6]. A Japanese pre- and postintervention study of an ambulatory regional palliative care intervention showed a small positive effect on quality of life in subgroups of cancer patients with poor general condition and patients without cancer treatment [7]. Further they found a decrease in perceived necessity for improvement of physical care provided by physicians and/or nurses, coordination/consistency of care and psychoexistential care.

General practitioners (GPs) play a decisive role in end-of-life care, not only because of their accessibility [8]. They are the first contact for patients and caregivers, particularly in the case of an incurable, progressive illness as especially in the case of circumstances which lead to less intensive medical care [9]. Many patients and their relatives see the GP as the ideal key worker in palliative home care [10]. A Belgish study showed that many terminally ill patients think that GPs have a very important role in continuity of relation and continuity of information [11]. Given these expectations, the self-perceived role of GPs in end-of-life care is most relevant and merits further evaluation.

### Current situation regarding end-of-life care provided by general practitioners in Germany und Switzerland

In Switzerland and in Germany there are coordinated, comprehensive palliative care programs currently being implemented nationwide. In Germany a change in legislation in 2007 established the rights to specialised palliative home care (Spezialisierte ambulante Palliativversorgung SAPV) for all patients who require it. It is offered by so called “specialist palliative care teams” which has specialist trained GP members as so called “palliative doctors” in it [12]. In Switzerland the Palliative Care Strategy sets similar priorities [13]. Due to the federal structure of both countries the level of implementation of the care programs varies considerably from region to region [12], [14], [15]. Due to the increasing complexity of treatment and care, the tasks of professional caregivers providing specialised palliative care in Switzerland can be understood to be approximately the same as those of the German SAPV [12], [16].

Clinical guidelines for identification of palliative patients in primary care are not currently in use either in Germany or in Switzerland [17]. From the perspective of terminally ill patients the GP is considered to play an important role in the process of care [8], [18–20]. However, until now there is still little knowledge about the end-of-life care provided by GPs, the actual frequency of dying and palliative care patients amongst GPs patients and the self-perceived role of family physicians regarding end-of-life care [21].

This study examines the scope of end-of-life care provided by GPs in southern North Rhine Westphalia (NRW) in the west of Germany and in the Canton of Bern in Switzerland.

## Methods

In April and May 2013 a two-page questionnaire was sent out by mail to active GPs. Our aim was to answer the following questions:

- What forms of symptom control were necessary for these patients?
- What other professional caregivers do GPs involve in their care and how often?
- How satisfied were the GPs with the care of their patients?
- Did the GPs consider themselves playing an important role?

In this paper, the author’s definition of a ‘palliative patient’ is a dying person in the last months of life. Palliative care means all aspects of care for palliative patients.

The requirement for research ethics approval was waived by the local research ethics committee (Kantonale Ethikkommission Bern) and the ethics committee of the Faculty of Medicine at the University of Bonn (no. 242/12). The requirement for research ethics approval was waived by the local research ethics committee (Kantonale Ethikkommission Bern).

### Surveyed regions

This study was conducted in two study regions. The first study region is in Germany and the second in Switzerland. First surveyed region: The standardised questionnaires were sent to all doctors teaching at the Departments of Family Medicine at the Universities of Cologne, Bonn and Aachen in southern NRW in the west of Germany. Additionally the questionnaire was also sent to a group of general practitioners and internists working in the city of Bonn in Germany, who had been identified via an online search (total 622). Second surveyed region: For Switzerland 579 GPs who were members of the Bern Society of Family Doctors and pediatricians were contacted. The Swiss GPs were questioned online only with the same questions.

The group of doctors in Germany initially received the questionnaire by mail. Three weeks later the group of teaching doctors in both study regions received the same questionnaire again in an online survey generated by “Survey Monkey” [22]. After data collection and digitalisation the data were aggregated.

### Questionnaire

The 2-sided questionnaire focusses with 11 closed questions on the physician’s most recently deceased patient. Physicians were asked to document the following patients’ characteristics: age, sex, cause of death, care requirements (medical, nursing, psychosochial, spiritual), symptomatology using the Minimal Documentation System MIDOS, a validated measure for self-assessment of pain and other symptoms in palliative care [23] (10 symptoms listed with the option to mark 1. Yes, low intense, 2. Yes middle intense, 3. Yes, high intense, 4. No or 5. Don’t know), participating care providers (11 options plus “Other: …”), as well as hospital treatment, emergency responses and home visits during the last phase of the patient’s life.

The physicians were also asked to assess the palliative care requirements of the deceased patients and to describe whether palliative care was necessary, whether it was available and how satisfied the doctors were with the different dimensions of care provided for the patient; i.e. whether or not the palliative care provided was considered to have been successful. This was to be answered on a 5-point Likert scale (ranging from very satisfied to very dissatisfied) with an additional “don’t know” option.

The physicians were also asked to name other services involved in the care of the patient. In both versions most of the questions are similar in content. At the end of the German questionnaire two definitions were stated relating to the special palliative care system. The first item explains the general palliative outpatient care and the second the specialized palliative outpatients care (see background section).

The length of the questionnaire was chosen so that it would take no more than ten minutes to complete. The data was transferred online or from the paper version into a common and aggregated electronic data bank.

### Statistics

The data collection and evaluation took place in anonymised form using IBM SPSS Statistics 20®. The descriptive and comparative data analysis was supervised by the Institute for Family Medicine in Bonn. Comparative analysis of the data collected in Germany and in Switzerland was made using the nonparametric chi-square testing method according to Pearson or, in the case of a comparison between two groups (Germany and Switzerland) on metric characteristics such as age, using the t-test for independent samples with normal distribution. The significance was set at *p* < 0.05.

For the evaluation of symptom burden a sum score was calculated out of the ten categorical symptom variables which had been collected, with symptom intensity ranked from 0 = absent to 3 = severe), so that the maximum rank-sum was 30. The rank-sum score is shown together with the standard deviation (SD).

## Results

Of the 1,201 general practitioners (GPs) contacted, 27.5 % (*n* = 330) sent back a filled-in and evaluable questionnaire. In Switzerland 140 questionnaires were returned (24.2 %) and in Germany 190 (30 %).

### Characteristics of the physicians surveyed

The average age of responding physicians was 54.5 years (range: 34–76; SD 7.4), 68 % of them were male and 45 % worked alone in their practice. The German GPs (average age 53.5 years, SD 7.5) were younger than those from Switzerland (average age 55.8 years, SD 7.1 – *p* = 0.005). The German group included significantly less men (62.4 %) than the Swiss group with 75.7 % (*p* = 0.01). With regard to the type of practice (single or group) there were no significant differences.

An absolute majority considered themselves to be indispensable to the care of palliative patients (76 % of the physicians surveyed in Germany and 91 % of those surveyed Swiss).

### Information on most recently deceased patients

The most recently deceased patients were on average 78 years old and 50.3 % were males (see Table 1). The most frequent cause of death was tumour disease, followed by cardiovascular disease. Evaluation of place of death and cause of death showed significant differences between the two regions in the survey. In Germany a much larger percentage of patients (43.2 %) died at home, compared with 27.1 % in Switzerland (*p* < 0.01), and in Germany far fewer people (24.4 %) died in nursing homes than in Switzerland (41.5 %; *p* < 0.05). It means that nearly 68 % of the patients died in domestic surroundings (at home or in a residential home), which form the GPs direct field of care.

**Table 1:** Characteristics of the most recently deceased patients

	S. NRW^b^ *n* = 190	Bern *n* = 140	Total *n* = 330	Group comparison
Average age (SD)	76.8 (13.5)	79.4 (15.9)	77.95 (14.7)	*p* > 0.05
Sex: male	51.1 %	49.3 %	50.3 %	*p* > 0.05
Place of death (in %)				
At home	43.2 %	27.1 %	36.4 %	value 26.92 (5 df^a^)
Nursing home	24.2 %	41.4 %	31.5 %	
General hospital	17.9 %	23.6 %	20.3 %	*p* < 0.01
Inpatient hospice	8.4 %	0 %	4.9 %	
Palliative care unit	4.2 %	4.3 %	4.2 %	
Other/not specified	2.1 %	3.6 %	2.7 %	
Cause of death (in %)				
Tumour	51.1 %	37.9 %	45.5 %	value 14.2 (5 df^a^)
Cardiovascular disease	18.9 %	32.9 %	24.8 %	
Infection	5.3 %	10.7 %	7.6 %	*p* < 0.05
Other	14.2 %	11.4 %	13 %	
Uncertain/inconclusive	8.4 %	5.7 %	7.2 %	
Not specified	2.1 %	1.4 %	1.8 %	

^a^ *df* = degrees of freedom; ^b^ *S.NRW* = southern North Rhine-Westphalia

The GPs surveyed in Germany reported more deaths from tumours (51.1 %), than their Swiss colleagues (37.9 %; *p* < 0.05).

### Symptoms in the end-of-life phase

The prevalent intensity of symptoms estimated by the GPs to have affected their most recently deceased patient is listed in Table 2. Nausea, emesis and constipation were rarely documented as having a high intensity in contrast to the other symptoms as you can see in Table 2. The mean symptom rank-sum was calculated to be 13.1 (SD 6.1).

**Table 2:** Symptoms of most recently deceased out-patients in the end-of-life period reported by GPs

	No symptoms	Symptoms with low or medium intensity	Symptoms with high intensity
Pain	24.2 %	54.0 %	16.5 %
Nausea	42.7 %	45.5 %	5.5 %
Emesis	67.3 %	21.8 %	3.3 %
Constipation	48.5 %	38.2 %	3.3 %
Shortness of breath	40.9 %	39.7 %	13.9 %
Lack of appetite	13.0 %	34.5 %	44.8 %
Tiredness	7.6 %	33.6%	53.0 %
Depressiveness	30.0 %	47.5 %	13.9 %
Fear	34.8 %	44.8 %	10.9 %
Weakness	4.8 %	25.2 %	65.5 %

### Care requirements of the most recently deceased out-patient

The need for medical care was documented by 89 % of the responding physicians. A need for nursing care was documented for 82 % of the deceased patients, 54 % required psychosocial support and 17 % spiritual support. Spiritual support was not defined in more detail in the study (whereby the authors understood it to mean all kinds of spiritual or religious support). There were no significant differences between the two surveyed regions.

### Care providers involved in end-of-life care

The patient’s family was involved in end-of-life care in 69 % of cases, 14 % of GPs documented involvement of a palliative care service and 5.2 % mentioned the involvement of volunteer services. Care was provided by a specialist palliative doctor for 32 of 186 dying patients (17 %) in Germany and in 18 cases (17 %) a specialist palliative care team was involved (Table 3).

**Table 3:** Care-providers involved in the end-of-life care

Care provider	Answers of the general practitioners (in percent)
General practitioner	86.1 % (*n* = 326)
Family	68.8 % (*n* = 326)
Care service	47.6 % (*n* = 326)
Chaplain	10.3 % (*n* = 326)
Palliative care service	13.9 % (*n* = 326)
Voluntary worker	5.2 % (*n* = 326)
Social worker	3.3 % (*n* = 326)
Specialist palliative doctor	17.2 % (*n* = 186) was clicked only in Germany
Palliative care team (PTC)	9.7 % (*n* = 186) does not exist as such in Switzerland

### Duties performed for the most recently deceased patient

Not only regular house visits but also emergency house calls or house calls for opioid therapy were documented for considerably more patients in the last week of life than in the preceding three months. At least one visit (regular or emergency) in the last three months of their life was required for 80.3 % of the patients, and 82.4 % were visited within the last year of their life (Table 4).

**Table 4:** Duties performed for the most recently deceased patient

Tasks performed at the end-of-life	In the last week^a^	In the last 3 months^a^	Most recently 3-12 months^a^
House visit (regular or emergency)	61.1 % (202)	80.3 % (265)	2.1 % (7)
Emergency calls	9.4 % (31)	17.3 % (57)	2.1 % (7)
Referral to hospital	17.9 % (59)	44.6 % (147)	6.7 % (22)
Opioid therapy	33 % (109)	51.2 % (169)	8.8 % (29)

^a^(count), *n* = 330

### Satisfaction of the physicians with different aspects of patient care

The GPs indicated a high degree of satisfaction in all areas (Table 5). More than half of the surveyed physicians gave no answer on spiritual care.

**Table 5:** Family doctors’ satisfaction with other care providers

	Very satisfied	Quite satisfied	Partly satisfied	Somewhat dissatisfied	Very dissatisfied	Don‘t know^a^
Psychosocial care	29.4 %	28.8 %	12.1 %	2.4 %	1.2 %	20.6 %
Symptom control	30.9 %	38.2 %	8.8 %	2.7 %	0.9 %	15.7 %
Nursing care	50.3 %	25.8 %	5.2 %	1.5 %	0.9 %	14 %
Spiritual care	11.5 %	13.9 %	7 %	1.5 %	0.6 %	56.7 %
Cooperation with all providers/coordination with other providers	39.7 %	26.7 %	8.8 %	4.2 %	1.2 %	16.1 %

^a^or no answer

The GPs evaluated the support given by themselves to family caregivers as being mostly “good” (42.7 %) or “very good” (33.9 %). The support for caregivers by other care providers is assessed by 42.1 % of the GPs as “good” and by 27.6 % as “very good”.

### Distribution of tasks in the care process

One question was to describe who undertook which tasks in the care process. In Switzerland the GP provided the psychosocial care in 54.3 % of the cases, compared to 60.2 % in Germany. A nursing care service was involved in 30.7 % of the Swiss cases, as opposed to 25.3 % in Germany. Symptom control was provided by the GP in 73 % of cases and by a nursing care service in 37.2 % (Germany) and 12.9 % (Switzerland) of cases. 37.2 % of the German doctors stated that a specialist palliative doctor was involved, whereas this was not documented at all in Switzerland.

In addition the German answers were evaluated for a correlation between the level of satisfaction of the GP and the care providers involved in symptom control. Satisfaction levels were the same when the doctors treated the symptoms themselves as when the treatment was carried out by other care providers.

According to the GP the nursing care was undertaken predominantly by a nursing care service (in 66.7 % of cases in Germany and 55 % in Switzerland). Here the GP was involved in only 8.6 % or 5 % of cases.

In Switzerland 10 % and 7.4 % of the German doctors provided spiritual care themselves. In both surveyed regions the coordination with other care providers was done in the majority of cases (71.5 % in Germany and 62.1 % in Switzerland) by the GPs themselves.

## Discussion

The age and the gender of the GPs surveyed reflect the German national average and correspond also to the statistics supplied by the Swiss Medical Association (FMH) for Switzerland [24, 25]. Because of the anonymisation and the low rate of return it is not possible to deduce whether the group of doctors whose answers were evaluated is representative of all GPs in the surveyed regions with regard to other characteristics such as the location of their office within the region.

### Recently deceased patients

Concerning the cause of death, the GPs most frequently documented cancer (45.5 %), which, according to federal statistics, is a higher percentage than the national average. In contrast cardiovascular diseases were less frequently documented as cause of death in our survey. According to the Federal Statistics Office in 2011 more than one in four deaths in Germany were caused by cardiovascular disease [26]. Cancer patients are thus over-represented in the care of GPs in this study, just as cardiovascular patients are under-represented. A possible explanation for this discrepancy is that cardiovascular patients may in more cases die in hospital in emergency care.

Many of the responding German physicians stated that they had special training in palliative care. This was not evident in Switzerland, where only few, non-standardised training opportunities in outpatient palliative care are available. But for Germany this difference may explain the significant prevalence of tumour patients reported, since cancer patients might have been referred specifically to GPs with a special interest in palliative care.

The comparison of the groups regarding place of death indicates a difference in the health care systems. There were significant differences between the numbers of patients dying in a care facility and those dying at home. After Iceland, Switzerland has the second highest percentage (6.6 %) of over 65 year-olds in residential care facilities, compared to Germany with 3.8 % [27]. This could explain the regional difference. A retrospective analysis of nearly 59,000 persons insured with the largest Swiss health insurance company over 5 years up to 2011 showed that 27 % died at home and 35 % in a residential care facility [28]. Of the Swiss patients reported in this study a similar percentage (27 %) died at home and slightly more (41 %) in a care facility. Overall nearly 68 % of the patients died in domestic surroundings (at home or in a residential home), which constitute the GP’s direct area of work.

### Symptom control

During the evaluation of the question on symptoms it became noticeable that symptoms such as nausea, emesis and constipation, which can be controlled more easily with medicines, were documented as having severe intensity less often than other symptoms. This indicates that the surveyed doctors are for the most part satisfied with the medical symptom control provided. This impression is confirmed by the answers to the question on satisfaction with treatment.

Worthy of note is the comparison between the symptom burden of the patients in this survey and patients in palliative care inpatient units. Data is available from patient self-assessment in the minimal documentation system MIDOS. This was used by Stiel et al. in 2009–2010 to ask patients during the hospital admission process to assess their own condition. The mean rank-sum score of 13.1 calculated in this study differs distinctly from the mean rank-sum score of the palliative in-patients referred to hospital by family doctors, which gave a figure of 9.6 [29], personal communication Lukas Radbruch 2013. It may be that in their self-assessment the inpatients actually underestimated the intensity of their symptoms. Reduced general condition and disorientation were criteria for exclusion. By contrast, it can be assumed in this study that the GPs had a recall bias towards their difficult cases, i.e. patients requiring more complex treatment. This would produce an overestimation of the rank-sums. In addition the point in time of the surveys (before death versus after death, using prevalence data in our survey and incidence data in the MIDOS survey of Stiel et al.) and the point of view (physicians’ assessment versus self-assessment) could possibly lead to a distortion of the stated extent of symptom intensity.

Over 90 % of the GPs were able to retrospectively rate the different symptoms. This indicates that MIDOS might be a suitable assessment tool in palliative home care.

### Involvement of different care providers

A comparison between the two countries on the involvement of different care providers in end-of-life care is also fraught with problems due to the different structures of the health care systems. However, in both regions the GPs considered themselves to be most frequently involved, followed by family caregivers and nursing care services. The percentage of dying home-care patients in Germany who also made use of a specialist palliative care team corresponds at 10 % exactly with the estimation of the Federal Joint Committee (Gemeinsamer Bundesausschuss -GBA) regarding the need for specialised palliative home care (SAPV) [30]. The use of palliative care services seems to be low in our study. This can at least partially be explained by a relatively low supply of palliative care services. As we mentioned above, the structured buildups of palliative care services in Germany and Switzerland are only a few years old. According to the EAPC atlas 2013 (EAPC: European Association for Palliative Care), Germany and Switzerland had a total supply of palliative care services of 7.32 per million inhabitants and 7.89, respectively [31]. The United Kingdom for example offers totally 15.43 palliative care services per million inhabitants, Belgium 18.08 and the Netherlands 15.32. However, a selection bias (they are focused mainly on their own profession) could also have led to an underestimation of the involvement of other care providers, so that further studies should be performed to give an objective assessment of the involvement of all possible care providers. Moreover it leads unclear, if the not specifically stated providers (e.g. psychologists, geriatricians or pharmacologists) are really not often involved by GPs or only not mentioned at the “Other”-Item of this question.

### GPs opinion about his or her own role

The answers to the question on tasks performed in end-of-life care confirm that the GP considers his or her role there to be particularly important. More than 80 % of GPs see themselves as being indispensable in end-of-life care and just as many doctors also made home visits in this phase. Their engagement was intensified in the last months of life.

### Spiritual and psychosocial care

The spiritual care of dying patients is a topic which GPs tend not to discuss and in which they are rarely personally involved. Further studies are required to establish whether they lack information, have no clear opinions or whether they do not see any demand. It would be interesting to investigate the reasons for this and to evaluate a potential need for training on spiritual and psychosocial needs, and/or the improvement of cooperation with the appropriate providers of these dimensions of care.

In 2006 in a partly structured telephone survey of GPs in Lower Saxony, Schneider et al. concluded that family doctors see the greatest need for improvement in psychosocial care, rather than in pain therapy [32]. In contrast almost 60 % of the doctors in our survey are quite or very satisfied with the psychosocial care provided for their dying patients.

### Strengths and limitations

At first it is important to emphasize, that the present study provides an insight into GPs perception of the own role in end-of-life care which might differ from the objective relevance. Nevertheless it is important to get an insight into their perspective because of the high relevance of this professional group in end-of-life care.

As in any retrospective survey it must be assumed that respondents did not remember everything, or that more complex situations were recalled and thus specified more often in the questionnaire (recall bias). The physicians may remember the more extensive care needed in the end-of-life situation of a tumour illness more easily than those patients who died suddenly as a result of chronic cardiac failure.

It is also probable that physicians participating in the survey were generally more interested in end-of-life care than all GPs (selection bias).

A comparison between the two surveyed regions was also difficult because of the different ways of data collection and because no information was available on the location of the doctor’s office, may have been an imbalance between urban and rural distributions in the samples. Moreover, no information from patients and relatives, or from palliative care specialists, has been taken into account. However, as far as the surveyed attributes are concerned (age, gender, type of office), the participating doctors conform to valid national statistics, so that it may be assumed that they are a representative sample. This is one of the study’s clear strengths, together with the precise examination of the GP’s attitude towards an aspect of patient care in which they see themselves as playing an important role.

## Conclusion

The great majority of GPs consider themselves to be indispensable in end-of-life care. These findings confirm and underline previous publications on this subject. However, the modest level of cooperation with volunteer services indicates that there is room for improvement. The results of the study confirmed that the care of patients at the end-of-life is an important part of GP’s work. It should therefore play an important role within specialised training.

The GPs maintain close contact with the family members of their patients. Other surveys have demonstrated that families and physicians are satisfied with this. Furthermore, this survey delivers an important insight into GPs’ perception of their own role. These results should be verified and deepened in further prospective studies which include a direct evaluation of the quality of care from patients and their caregivers.

## Abbreviations

GBA: German Joint Government Committee
GP: General practitioner
i.e: Latin: id est (that is to say)
MIDOS: Minimal documentation system
SD: Standard derivation
